# Compartment elasticity measured by pressure-related ultrasound to determine patients “at risk” for compartment syndrome: an experimental in vitro study

**DOI:** 10.1186/s13037-014-0051-4

**Published:** 2015-01-24

**Authors:** Richard Martin Sellei, Simon Johannes Hingmann, Philipp Kobbe, Christian Weber, John Edward Grice, Frauke Zimmerman, Sabine Jeromin, Frank Hildebrand, Hans-Christoph Pape

**Affiliations:** Department of Orthopaedic Trauma, Aachen University Medical Center, Aachen, Germany; Department of Orthopaedic Trauma, Sana Klinikum Offenbach am Main, Offenbach am Main, Germany; Department of Orthopaedics and Trauma, Queen Alexandra Hospital, Portsmouth, UK; Helmholtz-Institute for biomedical engineering, Chair of medical engineering, RWTH Aachen University, Aachen, Germany

**Keywords:** Compartment syndrome, Intra-compartmental pressure, Non-invasive diagnostic, Elasticity measurement, Elastography

## Abstract

**Background:**

Decision-making in treatment of an acute compartment syndrome is based on clinical assessment, supported by invasive monitoring. Thus, evolving compartment syndrome may require repeated pressure measurements. In suspected cases of potential compartment syndromes clinical assessment alone seems to be unreliable. The objective of this study was to investigate the feasibility of a non-invasive application estimating whole compartmental elasticity by ultrasound, which may improve accuracy of diagnostics.

**Methods:**

In an in vitro model, using an artificial container simulating dimensions of the human anterior tibial compartment, intra-compartmental pressures (p) were raised subsequently up to 80 mmHg by infusion of saline solution. The compartmental depth (mm) in the cross-section view was measured before and after manual probe compression (100 mmHg) upon the surface resulting in a linear compartmental displacement (∆d). This was repeated at rising compartmental pressures. The resulting displacements were related to the corresponding intra-compartmental pressures simulated in our model. A hypothesized relationship between pressures related compartmental displacement and the elasticity at elevated compartment pressures was investigated.

**Results:**

With rising compartmental pressures, a non-linear, reciprocal proportional relation between the displacement (mm) and the intra-compartmental pressure (mmHg) occurred. The Pearson coefficient showed a high correlation (r^2^ = −0.960). The intra-observer reliability value kappa resulted in a statistically high reliability (κ = 0.840). The inter-observer value indicated a fair reliability (κ = 0.640).

**Conclusions:**

Our model reveals that a strong correlation between compartmental strain displacements assessed by ultrasound and the intra-compartmental pressure changes occurs. Further studies are required to prove whether this assessment is transferable to human muscle tissue. Determining the complete compartmental elasticity by ultrasound enhancement, this application may improve detection of early signs of potential compartment syndrome.

**Electronic supplementary material:**

The online version of this article (doi:10.1186/s13037-014-0051-4) contains supplementary material, which is available to authorized users.

## Introduction

Early and accurate diagnosis of acute compartment syndrome (ACS) is crucial for timing of fasciotomy. Delayed diagnosis is the most relevant determinant of poor and sometime devastating outcome [1-4]. The incidence of a compartment syndrome of the lower limb associated with fracture and blunt trauma varies from 3 to 17% [5]. Also, overlooked compartment syndrome is a major liability risk for the treating physician. Templeman et al. estimated the costs and lawsuits for awards after overlooked compartment syndrome at $ 280,000.00 each case [6]. The transient state within the pathological pathways is difficult to detect and in unsure, imminent cases of ACS we tend to promote early fasciotomy. Therefore improvements of sensible and secure diagnostics are still needed and may help to avoid delayed or unnecessary treatment by surgery.

The pathophysiology of the compartment syndrome is well described [3,4,7,8]. Diagnosis of an ACS by clinical assessment of the soft-tissue swelling is the most important determinant to proceed to surgery. But daily clinical routine work suggests a weak reliability of manual palpation [9]. Invasive pressure measurement continues to be the gold standard to objectify suspected ACS [8]. However, non-invasive assessments potentially allow optimized monitoring [10]. Over the last two decades non-invasive diagnostic principles have been implemented. Several authors showed promising results when investigating either the soft-tissue elasticity or the muscle perfusion. But heir reliability and level of evidence have to be investigated [11-13]. However, there is still a need for a reliable tool assessing the soft-tissue swelling to objectify the clinical findings.

Ultrasound elastography is introduced in the early 1990s [14,15], which allows to differentiate the mechanical properties of tissue by qualitative visual or quantitative measurements [16]. Over the last two decades this technique evolved into a real-time imaging of the distribution of tissue strain related to its elastic modulus. The most common technique of stress application is the strain (compression, real-time) ultrasound elastography [17]. The low-frequency compression on the soft tissue (e.g. breast, abdominal organs, muscle) is usually applied manually with a hand-held B-mode transducer. The resulting axial tissue displacement or strain provokes different echo sets before and after compression, which is visualised by different colours. Thus ultrasound elastography provides information on relative tissue stiffness compared with the adjacent and surrounding tissue within the image section. There is limited date available on the use of real-time elastography for skeletal muscle [18,19]. Niitsu et al. recently described in a feasibility study in healthy volunteers the potential measurement of muscle hardness calculating the “strain ratio” by the relative elasticity of the biceps muscle compared to that of the reference before and after exercise [18]. Whether this application can be used to determine compartment pressures in ACS has to be investigated.

Gershuni et al. determined the pressure-volume correlation of the tibial anterior compartment by ultrasound and demonstrated a volume increase of the compartment after exercise in the cross-section view [20]. Rajasekaran et al. demonstrated a significant increase in muscle compartment thickness in patients with chronic exertional compartment syndrome compared with control subjects after exertion using ultrasound [21].

The described pressure-volume correlation of the muscle compartment implies a potential correlation of the pressure and the compartment elasticity. Aim of our study was to prove the feasibility of a pressure enhanced ultrasound measurement for non-invasive assessment of decreasing compartment elasticity whilst rising compartmental pressures. We therefore hypothesize that there is a measurable correlation between compartment pressure and its elastic behaviour, which may be transferable in a model of compartment syndromes aiming at early detection of increased or rising intra-compartmental pressures.

## Materials and methods

**Figure 1:**
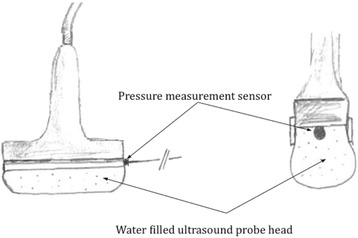
**The ultrasound probe connected with the water filled probe head containing the pressure sensitive measurement sensor.**

**Figure 2:**
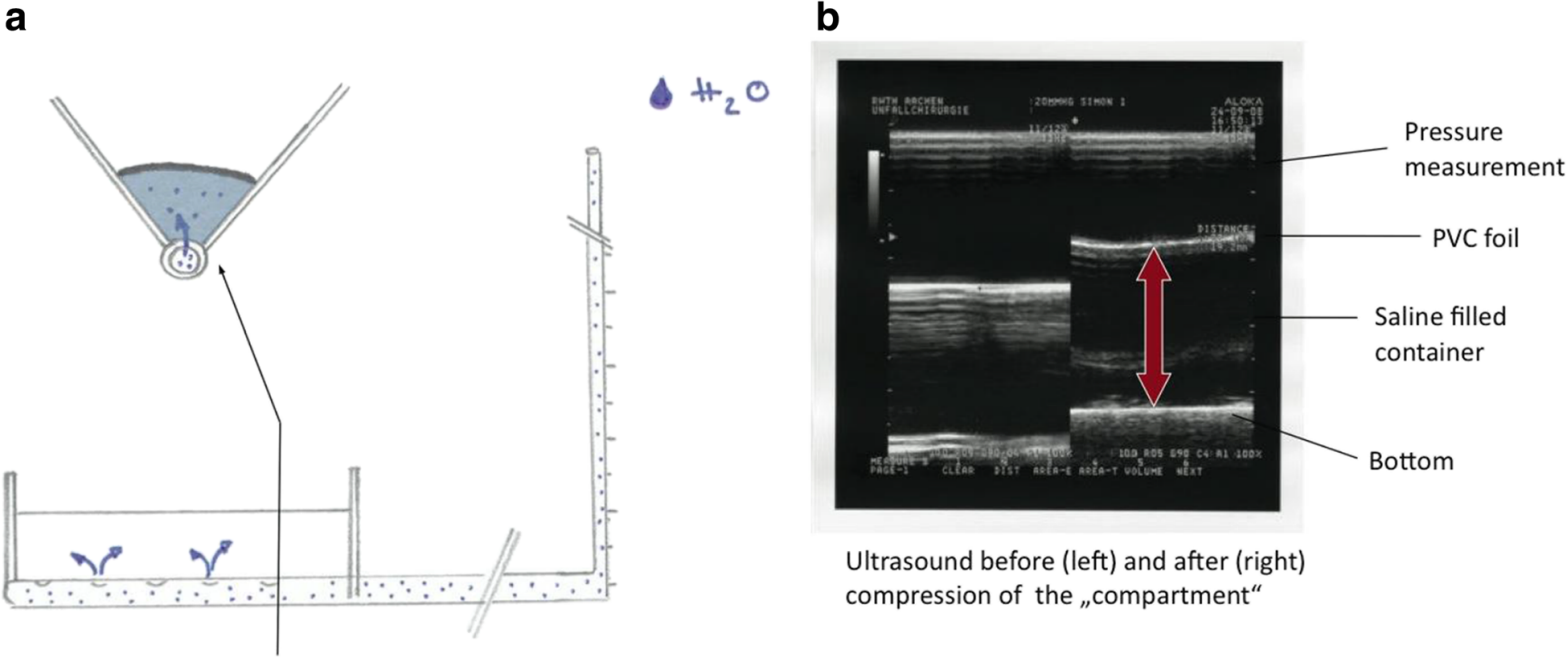
**In vitro model mimicking the dimensions of a human anterior tibial compartment filled with water, simulating razing pressures (2a).** Ultrasound cross-section view of the compartment model measuring the distance between the flexible foil (corresponding the fascia) and the compartment bottom **(2b)**.

A brightness-mode (B-mode) ultrasonic linear probe cross-section view for soft tissue imaging of a commonly used unit (Aloka SSD 1700, Dynaview II, Tokyo, Japan) was enhanced by a pressure-measuring device connected to a probe head (Figure 1). Therefore a pressure sensor was placed in the water filled cavity of the probe head and calibrated at barometric pressure. To investigate the relationship between the intra-compartmental pressure (ICP) and the linear compartment strain in the cross-section view (∆d), we built a container, filled with water, simulating the dimensions of a human male anterior tibial muscle compartment (30 cm × 6 cm). The walls are composed of compressed wood (thickness of 8 mm), whilst the bottom of the container was created by a perforated plastic tube (external diameter of 40 mm). The muscle fascia was approximated by an ultrasound conducting PVC-film (Polyvinyl-chloride film characteristics: 1.2 mm thickness, 22–25 N/mm^2^ tensile strength, 1.25-1.35 g/cm^3^ density). The pressure within this container was increased or decreased by an external water column, which was scaled to determine the pressure within the container (Figure 2a). This study has been performed in accordance with the ethical standards laid down in the 1964 declaration of Helsinki and has been approved by the appropriate committee of the local university.

### Measurement

The pressure sensitive transducer within the probe head determined the initiated manual low-frequency compression of the hand-held. Ultrasound images at 0 mmHg and at 100 mmHg probe pressure were taken. The thickness of the compartment (mm) corresponding the distance between the PVC-film and the bottom of the container was measured by using the ultrasound unit (Figure 2b). The difference between these two measures (Δd) was correlated to the pressure within the container. Filling up the water column, the pressure in the compartment increased. Measurements were obtained without locking the influent tube to allow elusion of the water into the column, simulating an elastic behaviour. The measurements were assessed stepwise at every 5 mmHg pressure increase. The results were figured in absolute and relative values.

### Statistics

The Pearson’s coefficient was determined to calculate the correlation between the simulated intra-compartmental pressure (ICP) and ∆d. Additionally, the intra-observer reliability was tested with ten measurements at ICP of 15, 30 and 60 mmHg each of the same observer. Inter-observer reliability (inter-rater agreement) was evaluated with ten different physicians, of various experiences, using the device to measure ∆d at the above mentioned ICP’s. They all were not involved into the study setup and construction to ensure the comparability of their results. The reliability was calculated using the Cohen coefficient (κ). Hereby, we excluded the inconclusive pressure measurement between 30 and 50 mmHg that imply re-evaluation. The probability level was set at p < 0.05. Statistical analyses were performed using MedCalc, version 11.3 (MedCalc Software, Belgium).

## Results

**Figure 3:**
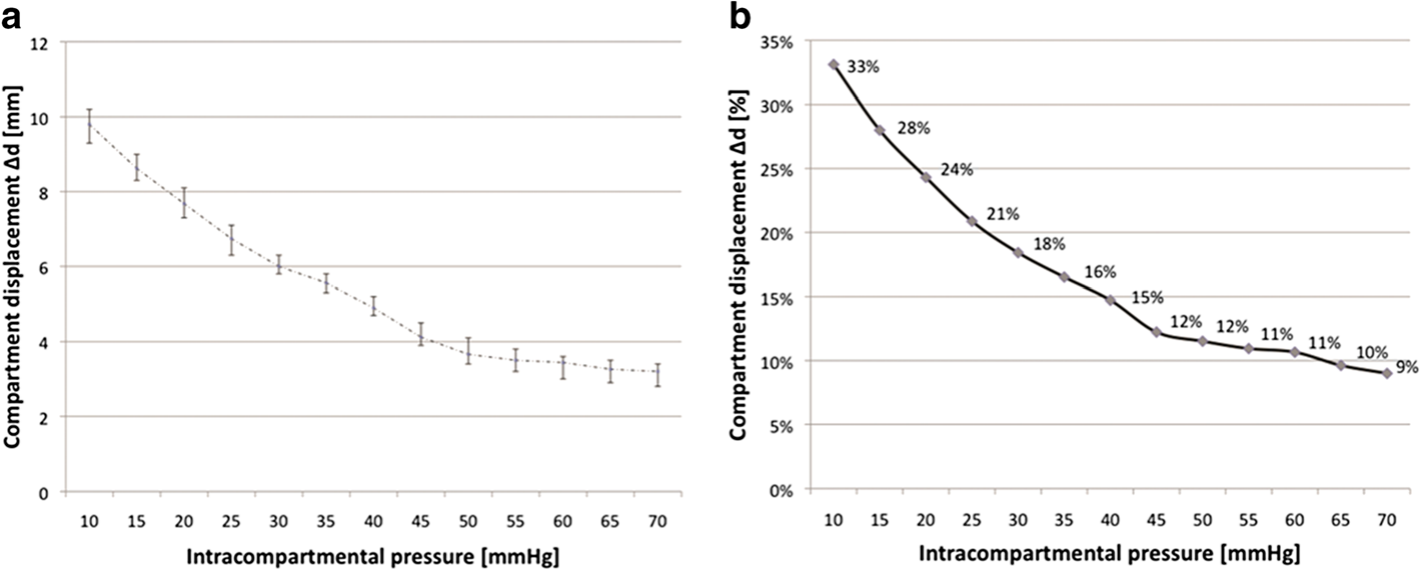
**Correlation between intra-compartmental pressure (p) and compartmental displacement (∆d) in the in-vitro model illustrated in absolute (3a) and relative values (3b).**

The in-vitro compartment model simulated an ICP between 0 mmHg and 80 mmHg. The assessment of the ultrasound cross-section view was used to measure ∆d. Each measurement at a specific level of ICP was repeated six times. The over all correlation, calculated with the Pearson’s correlation coefficient, revealed a value of r^2^ = − 0.958. The measurements and series were repeated to determine the reliability as described before. The model was free from any leakage at any the time of measurements. With rising compartmental pressures, an inversely proportional relation between ∆d and p occurred. The results are figured in absolute and relative values (Figure 3a,b). The intra-observer reliability, quantified with the Cohen coefficient kappa (κ), revealed a mean of κ = 0.840 (n = 10). This corresponds with a statistically very strong strength of agreement. Inter-observer reliability (n = 10) showed acceptable values (κ = 0.640). There was a statistically significant difference calculated between the ∆d at pressures less than 30 mmHg and greater than 50 mmHg (p < 0.005) using the Wilcoxon test to rank these values (Table 1).

**Table 1:** **Displacement of the compartment (∆d) in cases of physiological (≤30 mmHg) compared with pathological (≥50 mmHg) pressures (using the Wilcoxon test)**

**Interface motion within the compartment model (∆)**			
**(≤30 mmHg vs. ≥ 50 mmHg)**			
**Compartment**	**≤30 mmHg**	**≥50 mmHg**	**p-value**
	**mean values**	**mean values**	
Real motion [mm]	7.44	3.54	0.001
Rel. motion [%]	24.85	10.71	0.003

## Discussion

The invasive needle compartment pressure measurement remains the most valuable tool to assess and objectify elevated intra-compartmental pressures in uncertain cases. But its reliability, invasiveness, and interpretation of the thresholds have been discussed [22]. Hence, there is still a need for a reliable tool supporting the physician’s manual observation. Objectifying his interpretation of the affected compartment elasticity and its changes over time and several assessments may improve clinical diagnostics as well as avoid delayed surgery.

There is a close pressure-volume correlation described in the cross-section view of muscle compartments [20] and a significant increase in muscle compartment thickness was demonstrated [21]. This pressure-volume correlation of the muscle compartment implies a close correlation between the pressure and the compartment elasticity. Thus, our hypothesis was that there is a potential measurable correlation between compartment pressure and its elastic behaviour. We therefore measured the elasticity of the compartment assessed by its strain behaviour. The applied manual standardized compression force caused a displacement (=strain), which was expressed by ∆d (mm).

Our results show that pressure changes within the model revealed a measurable change of compartment strain. The use of ultrasound was feasible to detect the whole compartmental displacements after pressure application of the observer with the hand-held probe. The ultrasound pressure probe provides reliable data when used repeatedly.

As far as we know, this is the first pressure related measurement with an ultrasound probe head to determine compartment pressure and its complete strain behaviour.

There are important limitations of this study. The visco-elastic behaviour of human muscle compartment was momentously simplified in our model. Using a water filled cavity in our model the compartment behaviour (compared with the human muscle compartment) is reduced to a homogeneous pressure behaviour. This assumption is limited and questionable, because the anisotropic muscle stiffness properties are inhomogeneous and change during muscle activity and posttraumatic swelling. The most important property addressed in this model is the fact that the compartment pressure rises whilst compressed by the observer.

Our in vitro model is an approximation of the human compartment size. The compartment volume was not changed, simulating inter-individual discrepancy. Furthermore, the plastic foil does not have the natural elasticity of the human fascia, but seems to imitate its physical behaviour at different compartment pressures. Our model is not capable to tell anything about the clinical accuracy or reliability. It is unclear whether other factors such as compartment diameter, muscle tone, and surrounding tissue composition (subcutis size, adjacent bone surface, interosseous membrane) may confound these measurements. These limitations reduce the significance of the results and have an important impact on the conclusion of the transferability to clinical use, but reveal a potential feasibility of this technique. In summary a human subject model is needed to be more appropriate. However, the main purpose of our investigation was to test the feasibility of a simple but potentially useful technique, which may have a clinical use after further improvements.

The evidence of an improved accuracy in measuring the compartment elasticity is difficult to achieve. However, the demanded clinical relevance can neither be proven by an in-vitro model nor by a cadaver model in human subjects, simulating rising compartmental pressures. They all approximate the pathophysiology of compartment syndrome. The clinical relevance has to be shown in a prospective clinical trial comparing the clinical signs, the values of the invasive and non-invasive measurements.

We know that increased tissue pressure usually equilibrates throughout a muscle compartment [22,23,24]. But localized areas of increased tissue pressure within a compartment also may cause irreversible damage to muscle [25]. This pressure peak on the fracture, or blunt injury, site should be examined by the physician. The model presented in this study intends to simulate one fundamental property of compartment pressure behaviour: the rising resistance of the compartment whilst compressed externally. Trapped by the tight fascia the muscle compartment becomes a supposed homogeneous tube with rising pressures. Validating the soft-tissue and muscle firmness is one of the most important clinical finding when assessing the extremities in patients at risk of imminent or acute compartment syndrome [13,26,27]. These findings used to be related to the elasticity of the whole compartment. The compartment firmness is a direct manifestation of increased ICP and is probably the earliest objective sign of a compartment syndrome [5]. Thus, this should be recognised as an early parameter when changes in compartment elasticity occur. Assessing the elasticity (Young´s Modulus) of muscle tissue itself is challenging because of its anisotropic visco-elastic behaviour influenced by its pretension [28]. Therefore diagnostics of muscle elasticity by strain elastography [14] is a limited device to assess musculature and its pressure condition. But in cases of raised compartment pressures the elasticity of the whole compartment itself shows a clear correlation towards the compartmental pressures [20].

In literature there are innovative, non-invasive techniques proposed, aiming at improved assessment of soft-tissue concerning diagnose of an ACS. Either the blood perfusion of the musculature [10] or the intra-compartmental pressure [11,12] is in focus of these diagnostic devices. Their limitations vary in reliability, accuracy and feasibility.

However, there is still a need for optimizing the diagnostics of soft-tissue elasticity, especially in uncertain cases of imminent compartment syndromes [29]. Our hypothesis was confirmed by the results and showed, that a correlation took place between the simulated compartmental pressures and the measured changes of the compartment depth before and after the external manual compression. This behaviour reveals a possible use in non-invasive compartment monitoring.

## Conclusions

We investigated the relation between rising pressure changes of a liquid filled compartment and the decrease of elasticity detected by reduced displacement of the compartment diameter by ultrasound. This feasibility study demonstrates a possible correlation between the compartmental pressure and the displacement provoked by external compression. Despite the simplified in-vitro conditions, this technique may be transferable in a model of acute compartment syndrome aiming at early detection of increased compartmental pressures. However, “pressure related ultrasound” of the human muscle compartments may help to identify and to monitor patients “at risk” of potential compartment syndrome.
